# A Generic Multi-Compartmental CNS Distribution Model Structure for 9 Drugs Allows Prediction of Human Brain Target Site Concentrations

**DOI:** 10.1007/s11095-016-2065-3

**Published:** 2016-11-18

**Authors:** Yumi Yamamoto, Pyry A. Välitalo, Dirk-Jan van den Berg, Robin Hartman, Willem van den Brink, Yin Cheong Wong, Dymphy R. Huntjens, Johannes H. Proost, An Vermeulen, Walter Krauwinkel, Suruchi Bakshi, Vincent Aranzana-Climent, Sandrine Marchand, Claire Dahyot-Fizelier, William Couet, Meindert Danhof, Johan G. C. van Hasselt, Elizabeth C. M. de Lange

**Affiliations:** 1 Division of Pharmacology, Cluster Systems Pharmacology, Leiden Academic Centre for Drug Research, Leiden University, Leiden, The Netherlands; 2 Quantitative Sciences, Janssen Research & Development, a Division of Janssen Pharmaceutica NV, Beerse, Belgium; 3 Division of Pharmacokinetics, Toxicology and Targeting, University of Groningen, Groningen, The Netherlands; 4 Department of Clinical Pharmacology & Exploratory Development, Astellas Pharma BV, Leiden, The Netherlands; 5 Department of Medicine and Pharmacy, University of Poitiers, Poitiers, France; 6 Department of Anaesthesiology and Intensive Care Medicine, University Hospital Center of Poitiers, Poitiers, France; 7 Leiden University Gorlaeus Laboratories, Einsteinweg 55, 2333CC Leiden, The Netherlands

**Keywords:** blood-brain barrier, central nervous system (CNS), human prediction, pharmacokinetics, translational model

## Abstract

**Purpose:**

Predicting target site drug concentration in the brain is of key importance for the successful development of drugs acting on the central nervous system. We propose a generic mathematical model to describe the pharmacokinetics in brain compartments, and apply this model to predict human brain disposition.

**Methods:**

A mathematical model consisting of several physiological brain compartments in the rat was developed using rich concentration-time profiles from nine structurally diverse drugs in plasma, brain extracellular fluid, and two cerebrospinal fluid compartments. The effect of active drug transporters was also accounted for. Subsequently, the model was translated to predict human concentration-time profiles for acetaminophen and morphine, by scaling or replacing system- and drug-specific parameters in the model.

**Results:**

A common model structure was identified that adequately described the rat pharmacokinetic profiles for each of the nine drugs across brain compartments, with good precision of structural model parameters (relative standard error <37.5%). The model predicted the human concentration-time profiles in different brain compartments well (symmetric mean absolute percentage error <90%).

**Conclusions:**

A multi-compartmental brain pharmacokinetic model was developed and its structure could adequately describe data across nine different drugs. The model could be successfully translated to predict human brain concentrations.

**Electronic supplementary material:**

The online version of this article (doi:10.1007/s11095-016-2065-3) contains supplementary material, which is available to authorized users.

## Introduction

Central nervous system (CNS) drug development suffers from 91% attrition rate and especially the success rate in phase II is very low (1,2). The primary reasons for attrition are safety issues (3). Although the underlying physiological and pharmacological reasons for such failures are often not fully known they are likely related to a lack of knowledge or failure to account for a combination of on- and off-target site concentrations, target interaction and downstream signal processing. The first step in this cascade, obtaining quantitative insight into CNS target site concentration kinetics, is already a major challenge, and has been suggested as a major factor contributing to failure of novel drug candidates (4). During clinical drug development, typically only drug plasma concentrations are considered as marker for drug exposure, because quantifying drug concentrations in the brain is challenging. Hence, the ability to predict brain concentrations based on plasma data is highly relevant to further optimize CNS drug development.

The prediction of brain target site concentrations is controlled by several factors. First, the poorly penetrable blood-brain barrier (BBB) and the blood-cerebrospinal barrier (BCSFB) (5) limit passage of drugs from the systemic circulation into the brain. These barriers are associated with limited passive diffusion, and in addition various active transport and drug metabolism processes that systematically administered drugs need to pass. Second, the brain can be further subdivided into several distinct physiological compartments, including the brain extracellular fluid (ECF), brain intracellular fluid (ICF), and multiple cerebrospinal fluid (CSF) compartments. The specific disposition characteristics across these specific compartments further determines drug target site concentrations. Third, CNS drug target site concentrations are mediated by physiological flows including the microvascular blood flow, and brain ECF and CSF flows. Lastly, drug protein binding and the localized pH in specific sub-compartments further affect ultimate brain target site concentrations.

Passive drug transport processes are mediated through a combination of drug permeability properties, trans-membrane transport routes, and the surface areas of the BBB (SA_BBB_) and BCSFB (SA_BCSFB_) (5). Active drug transport is mediated by transport proteins such as P-glycoprotein (P-gp), multidrug resistance-associated protein (MRPs), organic anion transporters (OATs), and organic anion transporting polypeptides (OATPs). Even though the function and localization of these transporters has been extensively investigated in in-vitro and in-vivo studies, their precise functions is in some cases not fully understood (6).

Several experimental preclinical models have been developed to assess drug distribution to brain compartments. These models differ in terms of temporal and spatial resolution, and in their consideration of drug protein binding (7–10). For example, the combinatorial mapping approach has been recently introduced using unbound drug concentration with the brain slice technique (10,11). This approach can predict unbound drug CNS exposure at steady state in multiple brain compartments, but does not allow temporal characterization of drug concentration changes. Positron emission tomography (PET) is sometimes used also clinically, as a non-invasive imaging method to visualize spatiotemporal drug distribution in the brain. However, PET scan signals cannot distinguish parent compounds from their metabolites, or bound and unbound drug compounds in the brain (12). Finally, microdialysis allows serial sampling in multiple physiological compartments of unbound drug concentrations, hence is suited to characterize the time profile of drug concentrations in the brain (13).

In order to capture the time profile and complexity of interacting factors governing drug distribution across brain compartments as determined by microdialysis methods, mathematical modeling represents an indispensable tool. Specifically, physiologically based pharmacokinetic (PBPK) models are of interest, as these models aim to distinguish between system- and drug-specific parameters, allowing for translational predictions by scaling or replacing system- or drug-specific parameters from the rat to man (14). Several (semi-) PBPK models for CNS drug distribution have been published, with different levels of complexity (15–20). However, these models did not yet include validations of predicted human CNS concentrations (21). Recently, Gaohoa *et al* published a CNS PBPK model, which consists of four compartments such as brain blood volume, brain mass, cranial CSF and spinal CSF. This model was validated with human acetaminophen and phenytoin data. However, a limitation of this model is the lack of consideration of a brain extracellular fluid compartment (brain_ECF_), which is of critical importance for prediction of receptor binding kinetics for drugs acting on membrane bound receptors and ultimately drug efficacy (22).

Previously we have developed separate semi-physiological CNS PBPK models for three drugs based on microdialysis experiments in rats, which included unbound drug concentration-time profiles across multiple brain compartments (23–25). These models described the data well, but resulted in different individual model structures for each of these drugs.

The purpose of the current work was to develop a more generally applicable model structure that can be used to predict drug target site concentration-time profiles in human brain compartments based on rat pharmacokinetic (PK) studies. To this aim, we used published and newly generated datasets for a larger number of drugs, and we performed rigorous model validation on external datasets. Furthermore, the impact of key drug transporters was also included in our model. Finally, we investigated the performance of the developed model structure to predict human brain concentration-time profiles for acetaminophen and morphine.

## Materials and Methods

### Data for Model Development

An overview of experimental data for nine compounds with different physicochemical characteristics used for model development is provided in Table I. The physicochemical characteristics of the nine compounds are provided in Table SI. Data on 6 compounds were previously published, as indicated in Table I. For three compounds (paliperidone, phenytoin and risperidone), data were newly produced after single intravenous administration, as described below.

**Table I:** Summary of the Rat Brain Distribution Data for Model Development and External Validation

Study design		Model development										External validation	
		Published data							Newly produced data			Published data	Newly produced data
		Acetaminophen	Atenolol	Methotrexate	Morphine	Morphine	Quinidine	Remoxipride	Paliperidone	Phenytoin	Risperidone	Acetaminophen	Remoxipride
Species		rat	rat	rat	rat	rat	rat	rat	rat	rat	rat	rat	rat
Nr of animals		16	5	23	65	18	41	29	21	14	16	8	65
Dosage, mg/kg (infusion time, min)		16 (10)	10 (1)	40, 80 (10)	4, 10, 40 (10)	10, 40 (10)	10, 20 (10)	4, 8, 16 (30)	0.5 (20)	20, 30, 40 (10)	2 (20)	200^a^ (1)	0.7, 5.2, 14 (10)
Nr of samples (sampling times, min)	plasma	67 (0–240)	32 (0–120)	186 (0–300)	825 (0–360)	306 (0–190)	313 (0–360)	189 (0–240)	182 (0–360)	109 (0–480)	124 (0–360)	67 (0–180)	290 (0–240)
	dialysate	592 (0–240)	106 (0–120)	1065 (0–300)	238 (0–360)	299 (0–180)	1678 (0–360)	125 (0–240)	660 (0–240)	152 (0–480)	436 (0–240)	72 (0–180)	489 (0–240)
Active transport inhibitor		–	–	probenecid^b^	GF120918^c^	–	tariquidar^c^	–	tariquidar^c^	tariquidar^c^, probenecid^b^	tariquidar^c^	–	–
Dosage of active transport inhibitor, mg/kg (infusion time, min)		–	–	150 (10)	6 (cont)^d^	–	15 (10)	–	15 (10)	15 (10) 150 (10)	15 (10)	–	–
Data													
plasma		X	X	X	X	X	X	X	X	X	X	X	X
brain_ECF_		X	X	X	X	X	X	X	X	X	X	X	X
CSF_LV_		X		X			X						X
CSF_CM_		X		X			X		X		X		X
References		(6)	(69)	(25)	(70)	(71)	(24)	(26)				(72)	

*brain*_*ECF*_ a brain extracellular fluid compartment, *CSF*_*LV*_ a compartment of cerebrospinal fluid in lateral ventricle, *CSF*_*CM*_ a compartment of cerebrospinal fluid in cisterna magna

^a^; mg, ^b^; inhibitor of MRPs, OATs and OATPs, ^c^; inhibitor of P-gp,^d^; continuous infusion

For some of the drugs, active transport inhibitors were co-administered intravenously to characterize the effect of P-gp, MRP, OATs and OATPs, as indicated in Table I. The transport inhibitors included were probenecid as an inhibitor of MRPs, OATs and OATPs, and GF120918 or tariquidar as inhibitor of P-gp.

### Data for External Model Validation

For an external validation of the model, we used two separate rat datasets for acetaminophen and remoxipride, as indicated in Table I. The acetaminophen data was previously published, the remoxipride data was newly generated as described in the experimental section. For acetaminophen and remoxipride, two separate experimental datasets were available. For each drug, one of these datasets was used for model development, whilst the second dataset was used for external validation. The external validation with these second sets of data allows assessment of the robustness of our model predictions with respect to a different experiment and variation in experimental design.

### Animals

Animal study protocols were approved by the Animal Ethics Committee of Leiden University and all animal experiments were performed in accordance with the Dutch Law of Animal Experimentation (for approval numbers see Table SII). Male Wistar rats (225–275 g, Charles River, The Netherlands) were housed in groups for a few days (5–13 days) under standard environmental conditions with ad libitum access to food (Laboratory chow, Hope Farms, Woerden, The Netherlands) and acidified water. Between surgery and experiments, the animals were kept individually in Makrolon type three cages for 7 days to recover from surgical procedures.

### Surgery

Rats were anesthetized (5% isoflurane for induction, 1–2% as maintenance), and subsequently received cannulas in the femoral artery for serial blood sampling, and in the femoral vein for drug administration, respectively. Subsequently, microdialysis guides were inserted into different brain locations. The animals were allowed to recover for 1 week before the experiments were performed. One day before the experiment, the microdialysis dummies were replaced by microdialysis probes. For details on guides, probes and locations see Table SII.

### Microdialysis and Drug Administration

**Fig. 1:**
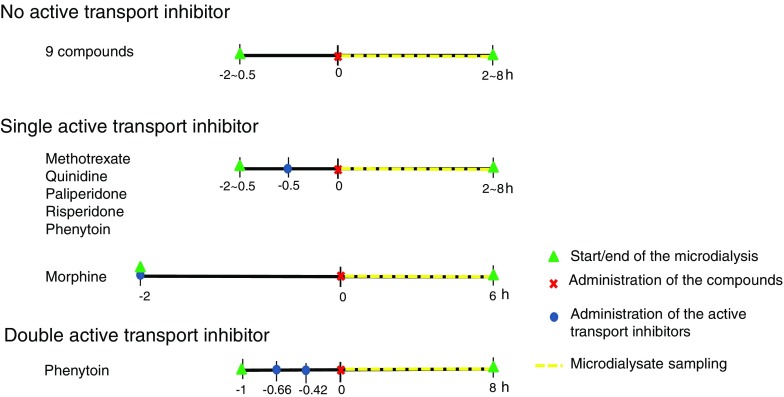
Microdialysis procedures for the compounds used for the development of the multi-compartmental brain PK model.

Experiments generally started at 9:00 a.m. to minimize the influence of circadian rhythms. Microdialysis probes were continuously flushed with microdialysis perfusion fluid (PF) until equilibration before the start of drug administration. Drugs were administered at *t* = 0 h by intravenous infusion through the cannula implanted in the femoral vein. For the quantification of active drug transport, the active transport inhibitor was administered before the drug’s administration. The general procedure of microdialysis is depicted in Fig. 1. Dosage and infusion time for each drug and the active transport inhibitor were summarized in Table I, and the composition of microdialysis PF and flow rate of microdialysis PF are summarized in Table SII.

### Bioanalytical methods

The developed analytical methods for risperidone, paliperidone, phenytoin and remoxipride are described below.

### Chemicals and Reagents

For all procedures, nanopure lab water (18.2 MΩ cm) was used. All chemicals used were obtained from Sigma Aldrich (Zwijndrecht, The Netherlands), and analytical grade unless stated otherwise. The internal standards risperidone-D4 and paliperidone-D4 were purchased from Toronto Research Chemicals (Toronto, Ontario, Canada). Remoxipride. HCl was obtained from TOCRIS (Bristol, United Kingdom). Tariquidar (TQD, XR9576) was obtained from Xenova group PLC (Cambridge, United Kingdom). Ammonium formate, ammonium bicarbonate (ULC/MS grade), acetonitrile (LC-MS grade), methanol, isopropanol, and formic acid (ULC/MS grade) were obtained from Biosolve B.V. (Valkenswaard, The Netherlands). Sodium hydroxide was obtained from Baker (Deventer, The Netherlands).

### Sample Preparation of Plasma

#### Risperidone and Paliperidone

The calibration curve was in a range of 5 to 1000 ng/ml. Quality controls (QC’s) were prepared in blank rat plasma at three different concentration levels and stored at −20°C. The lower limit of quantification (LLOQ) for both risperidone and paliperidone was 5 ng/ml. To 20 μl of plasma, 20 μl of internal standard solution (risperidone-D4 and paliperidone-D4) and 20 μl water (or 20 μl calibration solution in the case of the calibration curve) were added. After brief vortexing, 1 ml of acetonitrile was added. Brief vortexing and subsequent centrifugation at 10,000 g led to a clear supernatant, which was transferred to a glass tube and evaporated in the vortex evaporator (Labconco, Beun de Ronde, Breda, The Netherlands). The residue was redissolved in 200 μl of 2% methanol, 10 mM ammonium formate, pH 4.1 and processed in according to the solid phase extraction (SPE)- liquid chromatography (LC) method.

#### Phenytoin

20 μl of plasma sample was mixed with 20 μl of water in an Eppendorf vial. An aliquot of 40 μl acetonitrile was added for protein precipitation. After centrifugation at 11,000 g for 10 min, 40 μl of supernatant was mixed with 40 μl ammonium acetate buffer (pH 5.0). Calibration was performed by adding 20 μl of calibration solution to 20 μl of blank plasma, using the same clean-up procedure. The calibration solutions ranged from 0.2 to 100 μg/ml. 30 μl was injected into the high-performance liquid chromatography (HPLC) system. The LLOQ was 250 ng/ml.

#### Remoxipride

Sample preparation was performed according to Stevens *et al* (26). Briefly, 20 μl of sample was mixed with 20 μl of water and 20 μl internal standard (raclopride). Proteins were precipitated with 6% perchloric acid and centrifugation. After addition of sodium carbonate, 10 μl was injected into the SPE-LC system.

### Sample Preparation for Microdialysates

#### Risperidone and Paliperidone

The calibration curve for the microdialysis samples was prepared in buffered PF (composition in Table SII). The concentrations were in the range of 0.1 to 20 ng/ml. QC’s were prepared using a different batch of buffered PF. Before injection of 10 μl into the LC system, the microdialysate samples were diluted with internal standard solution in a ratio of 1:1 v/v. The internal standard solution consisted of 100 ng/ml risperidone-D4 and paliperidone-D4 in nanopure water. The LLOQs for risperidone and paliperidone were 0.4 and 0.2 ng/ml, respectively.

#### Phenytoin

Calibration curves were made in minimal PF at a concentration range of 25 to 5000 ng/ml. QC’s were prepared using a different batch of buffered PF. Of a typical sample that consisted of 40 μl of microdialysate, 30 μl was injected into the HPLC system. The LLOQ was 25 ng/ml.

#### Remoxipride

Calibration curves were prepared in buffered PF. The calibration range was from 1 to 200 ng/ml. QC’s were prepared using a different batch of buffered PF. Samples were mixed in a 1:1 v/v ratio with the internal standard raclopride (100 ng/ml) before injection of 5 μl into the LC system. The LLOQ was 0.5 ng/ml.

### Chromatography

#### Paliperidone and Risperidone

##### SPE-LC Method

For plasma samples the SPE-method was applied. The SPE system consisted of a Hyphere C8 HD, SE column (10 × 2 mm) (Spark Holland, Emmen, The Netherlands) in a cartridge holder and served for the clean-up of the sample. The cartridge holder was connected to a Gynkotek gradient pump (Thermo Scientific, Breda, The Netherlands) and a Waters 717 autosampler (Waters, Etten-Leur, The Netherlands). The MS Surveyor pump from Thermo Scientific (Breda, The Netherlands) provided the flow for the LC column, which was the same type as in the LC-method. The sample was injected onto the SPE, which was preconditioned with 2% methanol (pH 4.1). After 1 min of flushing, the SPE was switched into the LC system. After 4 min, the SPE was cleaned with 98% methanol (pH 4.1) for 2 min and reconditioned with 2% methanol (pH 4.1). The flow of the SPE pump was 0.75 ml/min. The flow of the LC system was 0.25 ml/min. The gradient was from 10 to 90% methanol (1–8.5 min after injection). The SPE column was used for a maximum of 240 injections.

##### LC-Method

For microdialysates, LC-Method was applied. The separation of the active compounds was possible using Hyper Clone HPLC column (3 μm BDS C18 130 Å) from Phenomenex (Utrecht, The Netherlands) placed at 40°C. The LC system was used at a flow of 0.25 ml/min using a linear gradient from 20 to 74% methanol (1–6 min after injection). Before the next injection, the column was re-equilibrated with 20% methanol for 2 min.

#### Phenytoin

##### HPLC Method and Detection

For both plasma and microdialysates samples an HPLC method was used. The mobile phase consisted of 15 mM ammonium acetate adjusted to pH 5.0 with acetic acid and acetonitrile in a 2:1 ratio (v/v). Separation was achieved using an Altima HP C18-Amide HPLC column (5 μm, 150 × 4.6 mm) from Grace Alltech (Breda, The Netherlands). The injector was from Waters (Etten-Leur, The Netherlands). The LC pump (LC-10 ADVP) was obtained from Shimadzu (‘s-Hertogenbosch, The Netherlands). The ultraviolet (UV) detector (Spectroflow 757) was obtained from Applied Biosystems (Waltham, Massachusetts) and was used at a wavelength of 210 nm. Data acquisition was achieved using Empower software from Waters (Etten-Leur, The Netherlands).

#### Remoxipride

##### SPE-LC Method

For the precipitated plasma samples, on-line SPE was combined with HPLC and mass spectrometry according to Stevens *et al* (26). Briefly, a pretreated sample was loaded into a Hysphere GP resin cartridge column (10 × 2 mm) from Spark Holland (Emmen, The Netherlands) at pH 8.3 and flushed for 1 min. Elution was performed using a low pH and an Altima HP C18 column (150 × 1.0 mm, 5 μm).

##### LC-Method

For microdialysates, a Kinetex 2.6 μm column (50 × 2.0 mm, XB-C-18) from Phenomenex (Utrecht, The Netherlands) was used at a flow of 0.6 ml/min and placed at 40 ^o^C. The system was a Nexera-*X*2 UHPLC system, consisting of two ultra high performance liquid chromatography (UHPLC) pumps delivering the high pressure gradient. A SIL-30 AC auto sampler was used to inject 5 μl of the microdialysis sample. The flow was diverted for the first 0.5 min, while a gradient from 10 to 90% methanol in 1.5 min served to elute both remoxipride and raclopride to the mass spectrometer.

### Mass Spectrometry

For risperidone, paliperidone and remoxipride, mass spectrometry was used to measure the concentrations. The mass spectrometer was a TSQ Quantum Ultra from Thermo Fisher Scientific (Breda, the Netherlands) and was used in MS/MS mode. Electrospray was used for ionization in the positive mode, nitrogen served as the desolvation gas and argon was used as collision gas. Data acquisition for both remoxipride and risperidone and paliperidone was performed using LCQuan 2.5 software from Thermo Scientific (Breda, The Netherlands).

Risperidone and paliperidone had the following transitions (m/z): 411.2→191.1 (risperidone), 415.2 →195.1 (paliperidone), 415.2 →195.1 (risperidone-D4), 431.2 →211.1 (paliperidone-D4). The scan width was set at 0.2 m/z, the scan time was 0.05 s. Collision was performed at fixed voltages between 27 and 38 V, using a skimmer offset of 2 V.

The transitions (m/z) were 371→242.8 for remoxipride and 247.0→84.0, 112, 218.8 for raclopride. The skimmer offset was 18 and collision was performed between at fixed voltages between 24 and 45 V. Scan width and scan time were the same as above.

### Determination of Fraction Unbound in Plasma

To determine the free fraction of paliperidone and risperidone in plasma samples, Centrifree Ultrafiltration Devices from Merck Millipore (Amsterdam, The Netherlands) were used to separate the free from the protein bound risperidone and paliperidone in pooled plasma samples. Both the ultrafiltrate and the original pooled plasma sample (without ultrafiltration step) were measured. The free fraction was calculated according to the following Eq. 1:

1 $$ Free\  fraction=\frac{Ultrafiltrate\  concentration}{Pooled\  plasma\  concentration} $$

For phenytoin and remoxipride, the free fraction in plasma was calculated using a protein binding constant of 91 and 26% respectively which were obtained from literature (27,28).

### Determination of In-Vivo Recovery (retro dialysis) (29)

The in-vivo recovery of paliperidone, risperidone phenytoin and remoxipride was calculated using the compound concentration in the dialysate (C_dial_) and in PF (C_in_) according to the following Eq. 2:

2 $$ In\  vivo\  recovery=\frac{C_{in}-{C}_{dial}}{C_{in}} $$

Brain microdialysis data of paliperidone, risperidone, phenytoin and remoxipride were corrected for in-vivo recovery to obtain brain_ECF_ and CSF data.

The in-vivo recovery and free fraction for the nine compounds are summarized in Table SII.

### Human Data

Table II summarizes the clinical concentration data for acetaminophen and morphine used to assess model performance to predict human concentrations. These data consisted of two clinical studies for acetaminophen and two studies for morphine. All studies were published, except for study 1 for acetaminophen that consists of newly generated data (see in Table II).

**Table II:** Summary of the Human Acetaminophen and Morphine Data

Study design		Acetaminophen		Morphine	
		Study 1	Study 2	Study 1	Study 2
Condition of patients		human with traumatic brain injury	human with nerve-root compression pain	human with traumatic brain injury	human with traumatic brain injury
Nr of patients		7	1 (mean values)	2	1
Dosage		1 g, 30 min infusion	2 g (propacetamol), short infusion	10 mg, 10 min infusion	10 mg, 10 min infusion
Nr of samples (sampling time, h)	plasma	38 (0–6 h)	11 (0–12 h)	23 (0–3 h)	11 (0–3 h)
	brain ECF or CSF	54 (0–5.5 h)	11 (0–13 h)	74 (0–3 h)	37 (0–3 h)
data references	Newly generated	(31)	(34)	(33)	
Data					
plasma		X	X	X	X
brain_ECF_				X (“normal” and “injured” brain tissue)	X (“normal” and “injured” brain tissue)
CSF_EVD_		X			
CSF_SAS_			X		
f_p_^a^		85%	85%	–	–
f_p_ references		(32)	(32)	(34)	(33)

*brain*_*ECF*_ a brain extracellular fluid compartment, *CSF*_*EVD*_ a compartment of cerebrospinal fluid in EVD, *CSF*_*SAS*_ a compartment of cerebrospinal fluid in subarachnoid space

^a^ free fraction in plasma

#### Acetaminophen

Acetaminophen human plasma samples and CSF samples were obtained at Poitier University Hospital. Seven patients who had a traumatic brain injury (TBI) were enrolled in the clinical study. They were treated with a 30 min intravenous infusion of 1 g of acetaminophen. CSF samples were collected from a compartment of cerebrospinal fluid in the lateral ventricle (CSF_LV_) by external-ventricular drainage (EVD) to control the intra-cranial overpressure (named CSF_EVD_) (30). All clinical studies were conducted according to the Declaration of Helsinki, and written informed consent was obtained from each subject after the approval of the institutional review board at the medical institute. The demographic data is summarized in Table SIII. Acetaminophen concentrations at the start of the study (some patients already received acetaminophen before) were used as an initial value in the plasma compartment. The volume of EVD samples and EVD flow rate during a certain time interval were experimentally determined (Table SIV).

A second human acetaminophen PK dataset (study 2) in plasma and in CSF subarachnoid space (CSF_SAS_) was obtained from the literature, and was based on patients with nerve-root compression pain (31).

For both datasets, total plasma concentrations for acetaminophen were converted to free plasma concentrations using the free fraction obtained from literature (32).

#### Morphine

Morphine human concentration-time profiles in plasma and in brain_ECF_ were obtained from the physiologically “normal” side of the brain and also from the “injured” side of the brain of TBI patients (33,34). For both datasets, the unbound plasma concentrations were already reported in the original publications (33,34).

### Software

The PK analysis was performed using NONMEM version 7.3 (ICON Development Solutions, Hanover, MD, USA) (35). For the brain PK modeling of rat data, the extended least squares estimation method was applied. Other analyses were performed by using the first-order conditional estimation method with interaction (FOCE-I). The compartmental models were defined using the ADVAN6 differential equation solver in NONMEM (35). The plots and the statistical analysis were conducted using R (Version 3.2.5; R Foundation for Statistical Computing, Vienna, Austria) (36).

### Model Development

Separate models describing plasma and brain concentration-time profiles for all nine compounds were developed whereby plasma- and brain-related parameters were estimated simultaneously. A naïve pooling approach was used (37), i.e. inter-individual variability in each compound’s data was not quantified, because of the highly standardized experimental settings combined with the homogeneous nature of the animals within each study.

The structural model that was used as a starting point was based on our previously developed models (23–25). To develop a more generally applicable model structure with parameters that can be precisely estimated across drugs, we systematically assessed the following two model structure characteristics.

First, a combined drug dispersion parameter was estimated to capture the CSF and ECF flow and turbulence flow of the drug molecules (38,39).

Second, drug transfer across the BCSFB was excluded. SA_BCSFB_ is 2–15 times smaller than SA_BBB_ (40–42), suggesting that drug exchange at BCSFB can be ignored from the model.

We evaluated for each drug the validity of the changes to the basic model with regard to a single or two different flow rates for drug dispersion and drug transport at the BCSFB.

### Quantification of Active Drug Transport

For the 6 compounds, data were obtained using co-administration of inhibitors of active transport. For all these compounds, the effect of the active transport inhibitors was tested on drug exchange at the BBB (Q_PL_ECF_) and plasma clearance (CL_PL_), and in combination, as a categorical covariate. (Eq.3)

3 $$ P={P}_{PAT}\times \left(1+{\theta}_{cov}\cdot Cov\right) $$

where P_PAT_ represents the parameter including passive and active transport (net transport), P represents the parameter which takes into account the active transport inhibitors if there is any such effect, Cov is the value of the covariate (0: without an active transport inhibitor, 1: with an active transport inhibitor), θ_cov_ represents the effect of the active transport inhibitor.

### Model Evaluation

The systematic inclusion of aforementioned factors was guided by a likelihood ratio test, by an adequate parameter estimation precision, by assessment of the parameter correlation matrix to ensure parameter identifiability, and by the graphical evaluation of plots for observations versus predictions and weighted residuals versus time and versus predictions. The likelihood ratio test is based on the assumption that changes in the NONMEM objective function values (OFV, -2 log likelihood) are asymptotically chi-square distributed. A decrease of OFV ≥ 3.84 was considered statistically significant (*p* < 0.05). For a clear assessment of model predictions and observations we also computed the following metrics (Eq.4 and 5).

4 $$ PE=\frac{Y_{OBS,ij}-{Y}_{PRED,ij}}{Y_{OBS,ij}-{Y}_{PRED,ij}/2} $$

5 $$ SMAPE=\frac{1}{N}{\displaystyle {\sum}_{k=1}^N\left|PE\right|\times 100} $$

where PE is a prediction error, and SMAPE is symmetric mean absolute percentage error (43). Y_OBS,ij_ is the *j*th observation of the *i*th subject, Y_PRED,ij_ is the *j*th prediction of the *i*th subject. N is number of observations. In the cases where we did not estimate inter-individual variability, e.g. for all brain PK data, Y_PRED,ij_ equals the mean population prediction Y_PRED,j_.

### External Model Validation

Validation of the brain PK model was performed by investigating the quality of the prediction of external rat data. The prediction was done as follows, 1) estimating plasma-related parameters (CL_PL_, Q_PL-PER1_ V_PL_ and V_PL_PER1_) using the external rat plasma data, 2) fixing the brain-related parameters (Q_PL_ECF_, Q_DIFF_, V_ECF_, V_LV_, V_TFV_, V_CM_, and V_SAS_) to the values which were estimated from the brain PK model and 3) predicting the brain_ECF_ or CSF concentrations using estimated rat plasma-related parameters and fixed brain-related parameters.

#### Plasma PK Analysis of External Rat Data

The plasma-related parameters including inter-individual variability on these parameters and residual errors were estimated using the external rat plasma data. We used a mixed effects modeling approach to investigate the predictability of the brain concentration based on each plasma concentration. The same plasma model structure, which was obtained from the brain PK model was applied for each compound. Inter-individual variability were tested on each PK parameter using an exponential model (Eq. 6).

6 $$ {\theta}_i=\theta \times {e}^{\upeta_{\mathrm{i}}} $$

where θ_i_ represents the parameters of the *i*th subject, θ represents the population mean value of the parameter, and η_i_ is the random effect of the *i*th subject under the assumption of a normal distribution with a mean value of 0 and variance of ω^2^.

A proportional error model and the mixed error model (Eq. 7-8) were tested for the residual errors:

7 $$ {C}_{ij}={Y}_{PRED,ij}\times \left(1+{\varepsilon}_{ij}\right) $$

8 $$ {C}_{ij}={Y}_{PRED,ij}\times \left(1+{\varepsilon_{1,}}_{ij}\right)+{\varepsilon_{2,}}_{ij} $$

where C_ij_ represents the *j*th observed concentration of the *i*th subject, Y_PRED,ij_ represents the *j*th predicted concentration of the *i*th subject, and ε_ij_ is the random effect of the *j*th observed concentration of the *i*th subject under the assumption of a normal distribution with a mean value of 0 and variance of σ^2^.

Model selection was guided by a likelihood ratio test with *p* < 0.05 and by the precision of the parameter estimates.

#### Handling of the Brain-Related Parameter Values

For Q_PL_ECF_, Q_DIFF_, the same values, which were estimated from the brain PK model, were used for acetaminophen and remoxipride, respectively. V_ECF_, V_LV_, V_TFV_, V_CM_, and V_SAS_ are system-specific parameters, therefore, the same rat physiological values were used, indicated in Table III.

**Table III:** Parameter Estimates for the Nine Compounds in Rat

		Parameter estimates (RSE, %)								
		Acetaminophen	Atenolol	Methotrexate	Morphine	Paliperidone	Phenytoin	Quinidine	Remoxipride	Risperidone
CL_PL_	mL/min	15.9 (4.80)	6.09 (6.80)	8.12 (3.90)	21.6 (2.60)	192 (7.80)	44.7 (5.40)	152 (2.40)	114 (2.70)	465 (13.0)
Q_PL_PER1_	mL/min	29.2 (19.9)	6.55 (12.2)	28.1 (18.0)	8.72 (3.80)	NA	133 (18.2)	1070 (5.80)	105 (10.7)	NA
Q_PL_PER2_	mL/min	NA	NA	1.50 (14.1)	53.3 (5.80)	NA	NA	NA	NA	NA
Q_PL_ECF_	mL/min	0.0281 (12.1)	0.00749 (15.6)	0.00109 (10.6)	0.00458 (7.40) 0.00750 (8.90)^d^	0.0123 (12.8)	0.00340 (13.7)	0.0354 (3.90)	0.0141 (8.90)	0.0247 (18.6)
Q_LV_PL_	mL/min	NA	NA	0.105 (10.6)	NA	NA	NA	NA	NA	NA
Q_ECF_ICF_	mL/min	NA	NA	NA	NA	0.0126 (21.0)	NA	0.0250 (6.70)	NA	NA
Q_DIFF_	mL/min	0.0556 (10.3)	0.0205 (11.7)	0.0598 (9.30)	0.0200 (4.30)	0.0248 (10.7)	0.0133 (13.0)	0.0237 (2.40)	0.0176 (8.20)	0.0254 (15.0)
V_PL_	mL	65.7 (25.4)	115 (12.3)	51.2 (20.3)	118 (6.40)	28400 (8.00)	2890 (7.90)	194 (37.5)	286 (16.6)	60000 (13.0)
V_PER1_	mL	219 (8.90)	280 (19.0)	210 (7.20)	1210 (7.80)	NA	5320 (7.80)	13300 (3.00)	2310 (6.40)	NA
V_PER2_	mL	NA	NA	114 (6.60)	570 (4.40)	NA	NA	NA	NA	NA
V_ECF_^e^ (53)	mL	0.29 FIX	0.29 FIX	0.29 FIX	0.29 FIX	0.29 FIX	0.29 FIX	0.29 FIX	0.29 FIX	0.29 FIX
V_ICF_^e^ (73)	mL	1.44 FIX	1.44 FIX	1.44 FIX	1.44 FIX	1.44 FIX	1.44 FIX	1.44 FIX	1.44 FIX	1.44 FIX
V_LV_^e^ (45,46)	mL	0.05 FIX	0.05 FIX	0.05 FIX	0.05 FIX	0.05 FIX	0.05 FIX	0.05 FIX	0.05 FIX	0.05 FIX
V_TFV_^e^ (74)	mL	0.05 FIX	0.05 FIX	0.05 FIX	0.05 FIX	0.05 FIX	0.05 FIX	0.05 FIX	0.05 FIX	0.05 FIX
V_CM_^e^ (48,49)	mL	0.017 FIX	0.017 FIX	0.017 FIX	0.017 FIX	0.017 FIX	0.017 FIX	0.017 FIX	0.017 FIX	0.017 FIX
V_SAS_^e^ (74,75)	mL	0.18 FIX	0.18 FIX	0.18 FIX	0.18 FIX	0.18 FIX	0.18 FIX	0.18 FIX	0.18 FIX	0.18 FIX
fraction	%	93.3(1.20)	NA	NA	NA	NA	NA	NA	NA	NA
Impact of active transport on										
Q_PL_ECF_		NA	NA	4.09 (3.7)^a^	1.62 (11.4)^b^	0.434 (11.4)^b^	0.355 (25.1)^b^	4.43 (2.80)^b^	NA	1.24 (16.3)^b^
Q_LV_PL_		NA	NA	0.410 (16.0)^a^	NA	NA	NA	NA	NA	NA
Standard deviations of residual error										
σ _plasma		0.341 (8.60)	0.218 (17.9)	0.522 (7.00)	0.647 (4.00)	0.631 (12.1)	0.444 (8.40)	0.418 (4.90)	0.348 (6.80)	1.44 (5.90)
σ _brain_ECF_		1.88 (6.70)	0.480 (17.8)	0.529 (6.20)	0.779 (7.40)	0.946 (6.80)	0.415 (7.00)	0.628 (3.30)	0.673 (6.80)	0.911 (6.80)
σ _CSF_LV_		0.607 (6.40)	NA	0.663 (5.70)	NA	NA	NA	0.629 (4.10)	NA	NA
σ _CSF_CM_		0.640 (8.20)	NA	1.00 (9.30)	NA	0.770 (7.60)	NA	0.466 (4.10)	NA	0.827 (9.40)

*CL*_*PL*_ clearance from the central compartment, *Q*_*PL_PER1*_ inter-compartmental clearance between the central compartment and the peripheral compartment 1, *Q*_*PL_PER2*_ inter-compartmental clearance between the central compartment and the peripheral compartment 2, *Q*_*PL_ECF*_ clearance from the central compartment to brain_ECF_, *Q*_*LV_PL*_ clearance from CSF_LV_ to the central compartment, *Q*_*ECF_ICF*_ inter-compartmental clearance between brain_ECF_ and brain_ICF_, *Q*_*DIFF*_ drug dispersion rate in brain and CSF, *V*_*PL*_ distribution volume of the central compartment, *V*_*PER1*_ distribution volume of the peripheral compartment 1, *V*_*PER2*_ distribution volume of the peripheral compartment 2,*V*_*ECF*_ distribution volume of brain_ECF_, *V*_*ICF*_ distribution volume of brain_ICF_, *V*_*LV*_ distribution volume of CSF_LV_, *V*_*TFV*_ distribution volume of CSF_TFV_, *V*_*CM*_ distribution volume of CSF_CM_, *V*_*SAS*_ distribution volume of CSF_SAS_, fraction; percentage of the drug which is reabsorbed by enterohepatic circulation, *RSE* relative standard error.

^a^; probenecid, ^b^; GF120918 or tariquidar, ^c^; dosage of 10 and 40 mg/kg, ^d^; dosage of 4 mg/kg, ^e^; physiological values

#### Prediction of brain_ECF_ and CSF Concentrations of External Data

Simulations were performed 200 times for each compound. The 95% prediction interval (using the calculated 2.5% tile and 97.5% tile) and the median of the simulated concentrations were plotted together with the external data. Accuracy of the mean population prediction for brain PK data was evaluated with SMAPE mentioned above (Eq. 5).

### Translation of the Model to Humans

**Fig. 2:**
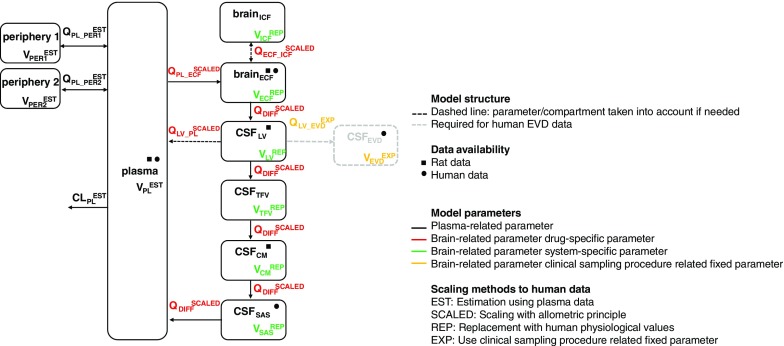
The brain PK model structure and translational methods for each parameter. The brain PK model consists of plasma, brain_ECF_, brain_ICF_, CSF_LV_, CSF_TFV_, CSF_CM_ and CSF_SAS_, which consists of 4 different categories parameters (*colors*). The scaling method on each parameter is indicated with color coding.

The translational prediction was performed by the following steps, 1) estimating plasma-related parameters (CL_PL_, Q_PL-PER1_ V_PL_ and V_PL_PER1_) using human plasma data, 2) replacing brain-related system-specific parameters (V_ECF_, V_LV_, V_TFV_, V_CM_ and V_SAS_) by human values, 3) applying allometric scaling to the brain-related drug-specific parameters which were estimated with the rat in-vivo data (Q_PL_ECF_ and Q_DIFF_), 4) adding clinical sampling procedure related fixed parameters which were obtained from the EVD into the model (Q_LV_EVD_ and V_EVD_) and 5) predicting the brain_ECF_ and CSF concentrations using estimated human plasma PK parameters, replacing system-specific parameters, scaling drug-specific parameters and using clinical sampling procedure related fixed parameters. The details of the translational methods for each parameter are explained in Fig. 2.

#### Human Plasma PK Analysis

Plasma-related parameters including inter-individual variability and residual errors were estimated using the human data using the Eqs. 6–8. A 1-compartment, 2-compartment and 3-compartment model were tested. Model selection was guided by a likelihood ratio test with *p* < 0.05, by the precision and correlation between parameter estimates and by the graphical evaluation of plots for observations versus predictions and weighted residuals versus time and versus predictions.

#### Replacement of the System-Specific Parameters

System-specific parameters in the brain distribution rat model (V_ECF_, V_LV_, V_TFV_, V_CM_ and V_SAS_) were replaced with the human physiological values, which are available from literature (44–50) (see Table IV).

**Table IV:** Parameter Values used for the Translational Prediction to Humans

	Translational methods	Unit	Parameter estimates (RSE, %)	
			Acetaminophen	Morphine
Plasma-related parameters				
CL_PL_	estimation from human PK data	mL/min	562 (20.1)	3070 (15.8)
Q_PL_PER1_	estimation from human PK data	mL/min	2060 (31.1)	3030 (0.60)
V_PL_	estimation from human PK data	mL	9880 (41.1)	16000 (35.3)
V_PER1_	estimation from human PK data	mL	51900 (18.3)	95400 (2.50)
Brain-related parameters				
Drug-specific parameters				
Q_PL_ECF_	allometric scaling	mL/min	1.92 FIX	0.513 FIX
Q_DIFF_	allometric scaling	mL/min	3.81 FIX	1.37 FIX
System-specific parameters				
V_ECF_^a^ (44)	replacement	mL	240 FIX	240 FIX
V_LV_^a^ (45–47)	replacement	mL	22.5 FIX	22.5 FIX
V_TFV_^a^ (45–47)	replacement	mL	22.5 FIX	22.5 FIX
V_CM_^a^ (48,49)	replacement	mL	7.5 FIX	7.5 FIX
V_SAS_^a^ (50)	replacement	mL	90 FIX	90 FIX
Clinical sampling procedure related fixed parameters				
Q_LV_EVD_	use the fixed parameter	mL/min	values are in supplemental Table IV	
V_EVD_	use the fixed parameter	mL		
Standard deviations of inter-individual variability (estimated from human PK data)				
ω_CL_PL_			0.490 (30.2)	0.271 (19.9)
ω_Q_PL_PER1_			NA	NA
ω_V_PL_			NA	0.596 (20.0)
ω_V_PER1_			0.235 (22.5)	NA
Standard deviations of residual error (estimated from human PK data)				
σ_plasma			0.250 (8.20)	0.0960 (22.9)

*CL*_*PL*_ clearance from the central compartment, *Q*_*PL_PER1*_ inter-compartmental clearance between the central compartment and the peripheral compartment 1, *V*_*PL*_ distribution volume of the central compartment, *V*_*PER1*_ distribution volume of the peripheral compartment 1, *Q*_*PL_ECF*_ clearance from the central compartment to brain_ECF_, *Q*_*DIFF*_ drug diffusion rate in brain and CSF, *V*_*ECF*_ distribution volume of brain_ECF_, *V*_*LV*_ distribution volume of CSF_LV_, *V*_*TFV*_ distribution volume of CSF_TFV_, *V*_*CM*_ distribution volume of CSF_CM_, *V*_*SAS*_ distribution volume of CSF_SAS_, *Q*_*LV_EVD*_ flow from CSF_LV_ to CSF_EVD_, *V*_*EVD*_ volume of CSF_EVD_

^a^; physiological values

#### Scaling of the Drug-Specific Parameters

Drug-specific parameters (CL_PL_ECF_ and Q_DIFF_) were scaled to human values using allometric principles following Eq. 9 (18).

9 $$ {P}_{human}={P}_{rat}\times {\left(\frac{B{W}_{human}}{B{W}_{rat}}\right)}^{0.75} $$

where P_human_ is the scaled human parameter, P_rat_ is the estimated rat parameter from the model, BW_human_ is the average human body weight (70 kg), and BW_rat_ is the average rat body weight (250 g).

#### Adding Clinical Sampling Procedure Related Fixed Parameters

In addition to those parameters which were used in the rat brain PK model, we have data obtained from the EVD approach, therefore the EVD compartment was added into the translated brain distribution model (see Fig. 2). To describe the PK of acetaminophen in the EVD compartment, the values of flow rate from CSF_LV_ to CSF_EVD_ (Q_LV_EVD_) and the volume of EVD compartment (V_EVD_) were added into the model. The values of Q_LV_EVD_ and V_EVD_ for each patient are obtained from EVD approach and available in Table SIV.

#### Prediction of Human brain_ECF_ and CSF Concentrations

Simulations were performed using the same methods as we mentioned for the external model validation.

## Results

**Fig. 3:**
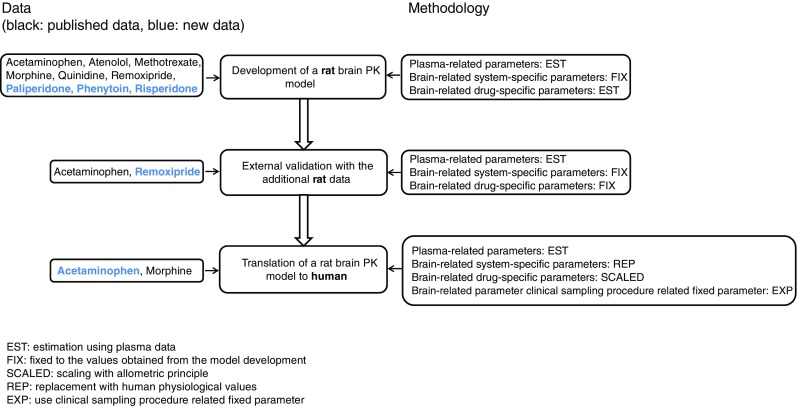
Schematic flow chart of the analysis.

**Fig. 4:**
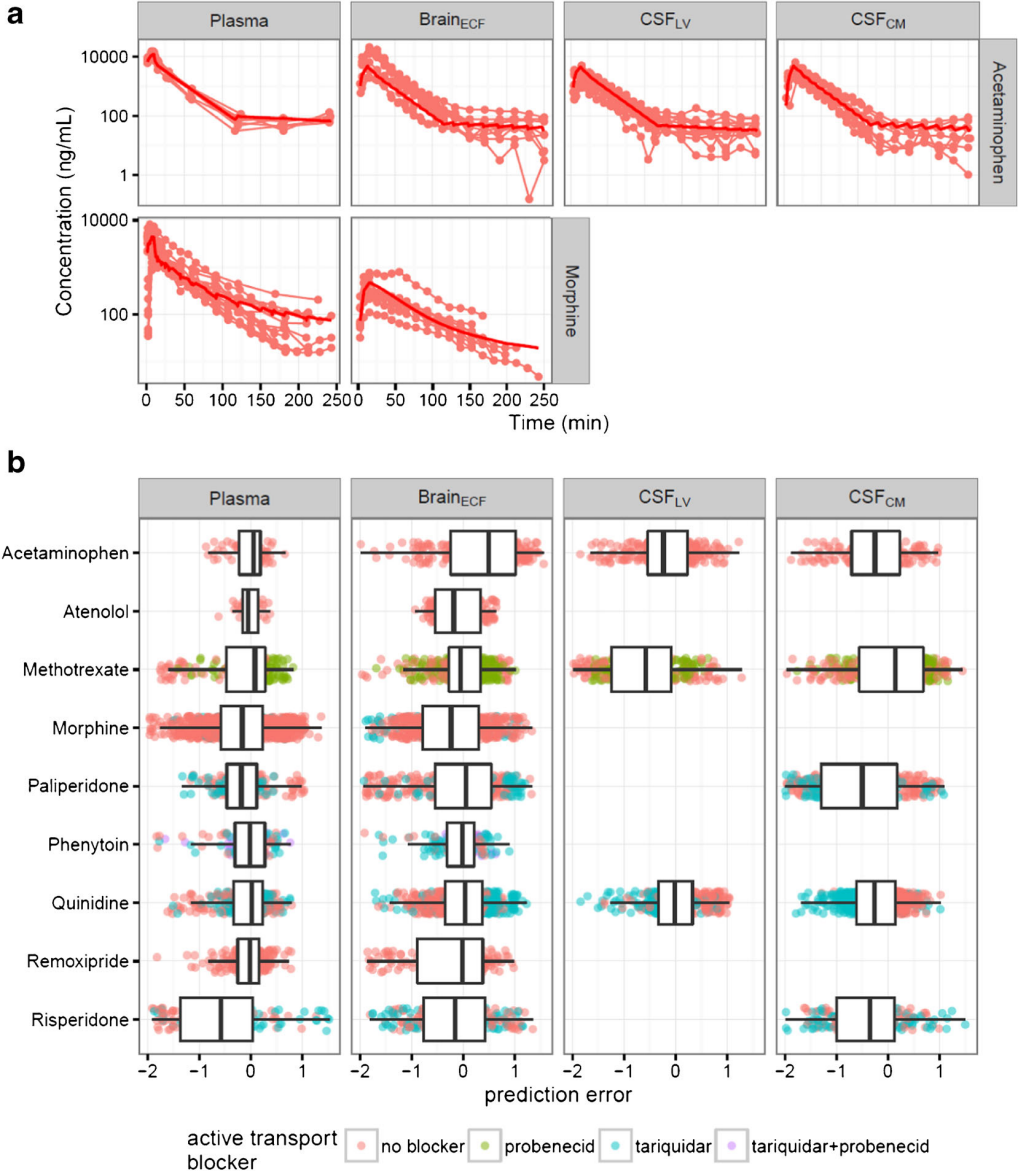
Prediction of the multi-compartmental brain PK model. (**a**) Individual observed drug concentrations (*lines and circles*) and mean model prediction (*solid lines*). Unbound concentration (ng/mL) versus time (min) profiles for acetaminophen and morphine. (**b**) Box-whisker plots for the prediction errors across all nine drugs evaluated. The plots were stratified by brain compartments (*panels*) and by active transport blockers (*colors*).

The analysis work flow is depicted in Fig. 3. The developed multi-compartmental brain PK model adequately described the data for the nine compounds, as can be observed from the selected observed and predicted concentration-time profiles (Fig. 4a) and the prediction error plots for all of the nine compounds (Fig. 4b). The prediction errors were mostly within two standard deviations of zero, i.e. no systematic differences between observations and predictions were found. No specific trend across time, also with respect to the presence or absence of active transport inhibitors, were observed. More extensive plots for individual observations versus predictions and weighted residuals versus time across drugs, dose levels and active transport inhibitors, are provided in the supplemental material (Figure S1 and S2).

We identified a generally applicable model structure (Fig. 2) with physiologically relevant compartments. The final model consists of plasma, brain_ECF_, brain intracellular fluid compartment (brain_ICF_), CSF_LV_, compartment of CSF in third and fourth ventricle (CSF_TFV_), compartment of CSF in cisterna magna (CSF_CM_) and CSF_SAS_, which included processes for drug exchange at the BBB (Q_PL_ECF_) and drug dispersion through brain_ECF_ and CSF compartments (Q_DIFF_). The parameter estimates were obtained with good precision, and are summarized in Table III.

A single drug dispersion rate (Q_DIFF_) was shown to be sufficient for describing the sum of the drug distribution in the brain_ECF_ and CSF for the nine compounds. Q_DIFF_ was comparable among the compounds, and ranged between 0.0598 mL/min for methotrexate to 0.0133 mL/min for phenytoin, and could be precisely identified (RSE < 15.0%), suggesting this parameter could be potentially considered to represent a system-specific parameter.

The parameter representing drug transfer at the BBB (Q_PL_ECF_) was critical to quantify drug exchange between blood and brain. Q_PL_ECF_ was substantially different between drugs, ranging from 0.0354 mL/min for quinidine to 0.00109 mL/min for methotrexate.

On the other hand, drug exchange at BCSFB was identified only for methotrexate, and could not be identified for the other 8 compounds. For methotrexate, the efflux transport at BCSFB (Q_LV_PL_) was 0.105 mL/min.

Among the nine compounds, clearance between brain_ECF_ and brain_ICF_ (Q_ECF_ICF_) could be estimated for paliperidone and quinidine: Q_ECF_ICF_ is 0.0250 mL/min for quinidine, and 0.0126 mL/min for paliperidone, implying for quinidine a slightly faster uptake into brain_ICF_ after crossing the BBB (Table III).

For morphine, brain_ECF_ concentration displayed a nonlinear relationship with dose and plasma concentrations. A categorical dose effect was therefore estimated. Continuous linear or nonlinear concentration-dependent effects to account for this effect were not supported by the data.

No statistically significant impact of P-gp and the combination of MRPs, OATs and OATPs on CL_PL_ could be identified, whereas those transporters were identified to act as efflux transporters at the BBB for our compounds. The P-gp function was quantified on the data of morphine, paliperidone, phenytoin, quinidine, and risperidone, and the impact of the combination of MRPs, OATs and OATPs was quantified on the data of methotrexate, as a categorical covariate on Q_PL_ECF_. The presence of P-gp inhibitors increased the Q_PL_ECF_ values of morphine, paliperidone, phenytoin, quinidine, and risperidone by 162, 43.4, 35.5, 443 and 124% respectively. The presence of the inhibitor of MRPs, OATs and OATPs increased the Q_PL_ECF_ values of methotrexate by 409%.

**Fig. 5:**
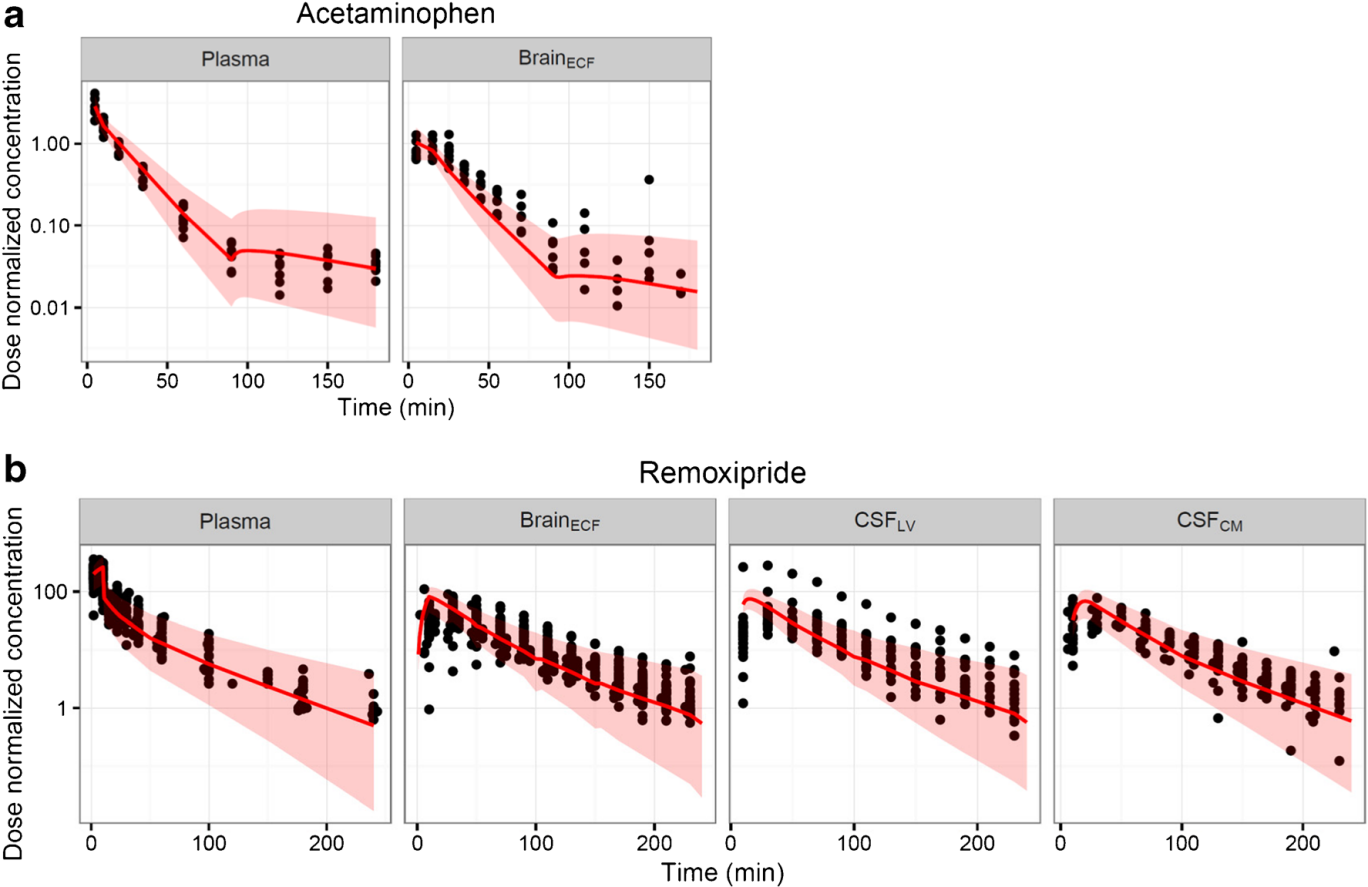
Model prediction versus external acetaminophen and morphine data in rat. Individual concentration-time profile of the external data (*circles*) and prediction from the brain PK model (*red lines*: median, shaded area is 95% prediction interval). (**a**) Acetaminophen data were obtained after 200 mg administration, (**b**) remoxipride data were obtained from the dose group of 0.7, 5.2 and 14 mg/kg. The *x-axis* represents the time in minutes and the *y-axis* represents the dose-normalized acetaminophen and remoxipride concentration. The panels are stratified by brain compartments and compounds.

The developed model adequately predicted the external rat acetaminophen and remoxipride data. Figure 5 presents the prediction results for the external rat data of acetaminophen and remoxipride using the developed multi-compartmental brain PK model. Prediction of the acetaminophen concentration-time profile in brain_ECF_ using the final model captured the external acetaminophen concentration in brain_ECF_ well (SMAPE < 61%). Prediction of the remoxipride concentration-time profile in brain_ECF_, CSF_LV_ and CSF_CM_ using the final model also captured the external remoxipride concentrations in brain_ECF_, CSF_LV_ and CSF_CM_ concentrations well (SMAPE < 67, 77, 56%, respectively).

**Fig. 6:**
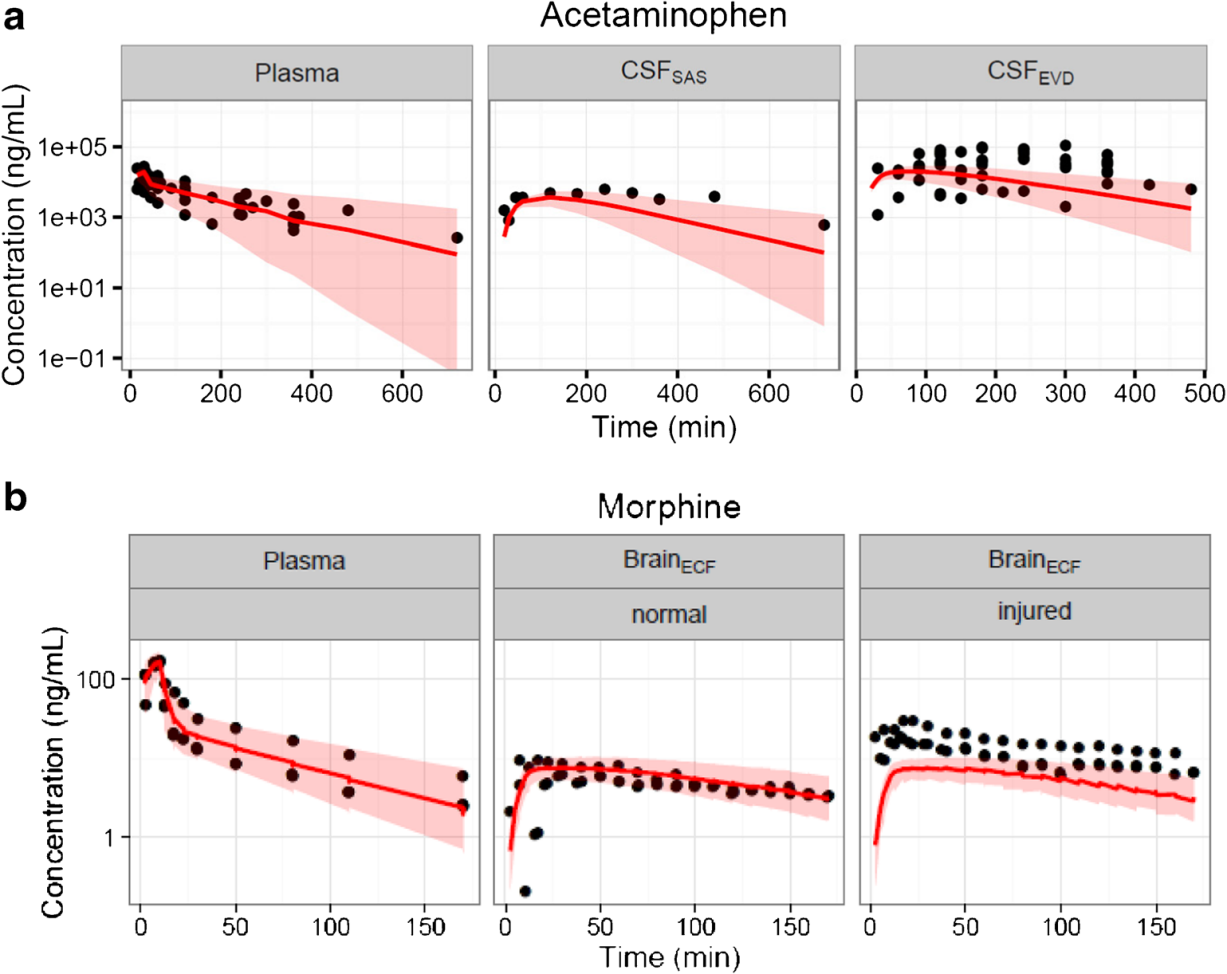
Human brain_ECF_ and CSF concentration-time profiles (*circles*) and prediction from the translational model (*red lines*: median, shaded area is 95% prediction interval). (**a**) Acetaminophen data was obtained from plasma, CSF_SAS_ and CSF_EVD_, (**b**) morphine data was obtained from plasma and brain_ECF_ in “normal” brain and “injured” brain. The *x-axis* represents the time in minutes and the *y-axis* represents the acetaminophen and morphine concentration in ng/ml. The panels are stratified by brain compartments and brain conditions.

The model was successfully scaled to predict concentration-time profiles of acetaminophen and morphine in human brain compartments. Table IV summarizes the parameter values that were used for the prediction of human plasma, CSF_EVD_, CSF_SAS_ and brain_ECF_. In Figure 6, the human predictions versus human observations are depicted. The acetaminophen human CSF_SAS_ concentration in the patients with nerve-root compression pain and CSF_EVD_ concentration in the patients with TBI were predicted relatively well (SMAPE < 90 and 66% respectively), even though there is a slightly faster elimination in CSF_SAS_. Morphine brain_ECF_ concentrations in the physiologically “normal” brain tissue of TBI patients were predicted very well (SMAPE < 35%). However, morphine brain_ECF_ concentrations were underpredicted when the brain_ECF_ concentrations were taken from “injured” brain tissue of TBI patients (SMAPE < 56%).

## Discussion

The developed multi-compartmental brain PK model could describe the data of the nine compounds in the rat adequately in the absence and presence of active transport blockers (Fig. 4). After scaling of the model, human brain concentration-time profiles of acetaminophen and morphine could be adequately predicted in several physiological compartments under normal physiological conditions.

The model structure we have derived differs from the ones published earlier by: (i) a combined drug dispersion parameter was estimated to capture the CSF and brain_ECF_ flow and turbulence flow of the drug molecules; and (ii) drug transfer across the BCSFB was excluded (23–25). The final model has four different CSF compartments. This model is developed to predict human brain concentration profiles using rat data. In our analysis, rat data was sampled from CSF_LV_ and CSF_CM_. Since in rats it is anatomically easier to access the CSF_CM_ compartment to obtain drug concentration by microdialysis and by the cisternal puncture methods, there are more data available from CSF_CM_ (51). Through keeping the CSF_CM_ compartment in the model structure, it will be easier to apply the model to additional compounds’ data obtained in animals. Furthermore, substantial differences between CNS compartments may exist, such as a concentration difference between CSF_LV_ and CSF_CM_ for methotrexate and quinidine in rat (24,25). Thus, to predict the drug target site concentration, the location of the CSF sampling site should be taken into account. For human, in clinical studies most CSF samples are taken from other CSF compartments, such as CSF_SAS_ and CSF_LV_ where samples are taken by EVD. Hence, we think that our model structure is a minimal, necessary model structure for translation.

We found that the brain intracellular fluid compartment (brain_ICF_) is required for the description of drug distribution of quinidine and paliperidone, and likely associated with the lipophilic basic nature of quinidine (pKa 13.9, log P 3.4) and paliperidone (pKa 13.7, log P 1.8). For other compounds with a less distinct lipophilic-basic nature, such as for acetaminophen and phenytoin, we have shown that brain_ICF_ was not required for the description of concentration-time profiles in the brain. However, for a generally applicable brain PK model, inclusion of this compartment would still be required since prediction of intracellular drug concentrations would be of relevance for CNS drug development as well as prediction of extracellular drug concentrations. Our model and the microdialysis methodology used only allow quantification of extracellular concentrations. However, in combination with PBPK modeling based principles to predict intracellular partitioning, our model will be of significant relevance as it provides the required predictions for unbound extracellular drug concentration kinetics.

A drug exchange parameter across the BCSFB (Q_LV_PL_) was identified for methotrexate only, even though it could not be identified for the other 8 compounds. This suggests that an additional efflux transporter might be present at the BCSFB for which methotrexate is a substrate. It is known that methotrexate is indeed a substrate of various transporters, such as RFC1, MRP, BCRP, OATP and OAT transporters (25), which are not involved in the drug transfer of the other 8 compounds. This result indicates that drug transport at BCSFB still needs to be investigated using data on compounds which are substrates for those transporters. The current model delineates the process that can be used to arrive to the best-performing model for such drugs. We took care to design the modeling process such that the total number of models that need to be fitted is minimal.

We identified a drug dispersion rate parameter that captures drug dispersion from brain_ECF_ to CSF. The median estimated drug dispersion flow was 0.0237 mL/min. The magnitude of the drug dispersion rate was approximately ten times faster than the reported physiological CSF flow rate alone (52), and about 100 times faster than the reported physiological brain ECF bulk flow rate (53,54). Since similar values across drugs were identified, the parameter may be considered a system-specific parameter that could be fixed in further analyses (see Table III), to allow for estimation of other processes of interest.

P-gp transport for quinidine, risperidone, paliperidone, morphine and phenytoin was confirmed as efflux transporter at the BBB which were in line with literature (55,56). P-gp transporter effects were not identified at the BCSFB for these 5 P-gp substrates, i.e. CSF concentrations for these compounds were well-described solely by the BBB mediated P-gp transport. The role and contribution of P-gp transporters at the BCSFB is still inconsistent, and both efflux and influx processes have been reported (57–59). Our results however suggest that the function of P-gp may be ignored, since its potential magnitude likely is negligible compared to transport at the BBB, and drug dispersion processes prevail. Nonetheless, overall, we envision that the combination of our dynamical modeling approach with the incorporation of in-vitro assays to characterize active transport across the BBB or BCSFB, may be a fruitful direction to further characterize and disentangle the precise contribution to the brain drug disposition of different drug transport.

The developed model adequately predicted the external acetaminophen and remoxipride rat data, confirming the reliability of the model. Both of these drugs were also used for model development, but the experiments were different and applied somewhat different designs. Since we aimed to generate mean predictions, the variation in numbers of animals is expected to result in limited bias in the modeling. Furthermore, sampling time points were very informatively distributed and any inter-experimental differences in these time points are therefore also considered to be of limited impact on model development. The external validation results indicated that the model is robust with respect to variations in experimental designs and conditions (i.e. the number of rats, sampling times, infusion times, and flow rates of microdialysis).

We consider the developed model structure suited for translational predictions of human brain (target site) concentrations such as required during drug development. The predictive performance in human data ranged between SMAPE of 35–90%. Even though errors <90% may appear large, such < two-fold error is not considered unacceptable when compared to for instance QSAR studies, which are used to predict unbound brain partition coefficients of drugs in drug development (60,61). Secondly, the prediction error is likely inflated because of the use of human data obtained from patients with traumatic brain injury or with nerve-root compression pain. Therefore, larger variability in their physiological condition is expected.

Body weight in combination with allometric scaling was used to scale the parameters to humans, and this resulted in adequate predictions of human brain concentrations for physiologically “normal” brains. Different translational methods for estimation of CNS PK parameters have been reported in the literature. For instance, system-based scaling was applied using volume of brain tissue or brain endothelial surface area (25,62), but allometric scaling using body weight (our approach) was supported by work from others in the literature (63–66). Based on our current approach, reasonable predictions were obtained. Therefore, we suggest that the allometric scaling approach may indeed be appropriate although it would be worthwhile to investigate alternative approaches.

Our model was developed based on healthy rats and then translated to human data that was partly based on patients with severe brain injuries. Indeed, observed human morphine concentrations in brain_ECF_ obtained from the “injured” side of the brain of the TBI patients was higher than the prediction from the translational model (Fig. 6). It is known that the BBB permeability is increased after TBI, which may be the reason for the under-prediction of our translational model for those data (67,68). Therefore, for predictions in patients with pathological conditions that alter the integrity of BBB or BCSFB barriers, or brain fluid flows, our model should be further extended with additional physiological details.

## Conclusion

A multi-compartmental brain PK model structure was developed across a wide range of drugs with different physicochemical properties. The model structure was shown to be of relevance for the scaling of brain concentrations in humans. As such, the developed model structure can be used to inform the prediction of relevant target site concentrations in humans and aid in the translational development of CNS targeted drugs.

## Electronic supplementary material

Below is the link to the electronic supplementary material.

Fig. S1 Model prediction (solid lines) versus observation (lines and circles) of the nine compounds in rat for each dose and without and with co-administration of active transport blockers. The x-axis represents the time in minutes and the y-axis represents the concentration of the nine compounds in ng/ml. The panel is stratified by brain compartments and by active transport blockers (colors). (EPS 532 kb)

Fig. S2 Weighted residuals versus time of the nine compounds in rat. The x-axis represents the time in minutes and the y-axis represents the weighted residuals of the nine compounds. The panel is stratified by brain compartments and by active transport blockers (colors). (EPS 263 kb)

ESM 3 (DOCX 33 kb)

ESM 4 (DOCX 38 kb)

ESM 5 (DOCX 34 kb)

ESM 6 (DOCX 40 kb)

## ABBREVIATIONS

BBB: Blood–brain barrier
BCSFB: Blood–cerebrospinal fluid barrier
brain_ECF_: Brain extracellular fluid compartment
brain_ICF_: Brain intracellular fluid compartment
CNS: Central nervous system
CSF: Cerebrospinal fluid
CSF_CM_: Compartment of cerebrospinal fluid in cisterna magna
CSF_EVD_: Compartment of cerebrospinal fluid obtained by external-ventricular drainage
CSF_LV_: Compartment of cerebrospinal fluid in lateral ventricle
CSF_SAS_: Compartment of cerebrospinal fluid in subarachnoid space
CSF_TFV_: Compartment of cerebrospinal fluid in third and fourth ventricle
ECF: Extracellular fluid
EVD: External-ventricular drainage
ICF: Intracellular fluid
IPRED: Individual prediction
PF: Perfusion fluid
PK: Pharmacokinetic
PRED: Population predictions
SA_BBB_: Surface area of blood–brain barrier
SA_BCSFSB_: Surface area of blood–cerebrospinal barrier
SMAPE: Symmetric mean absolute percentage error
SPE: Solid phase extraction
TBI: Traumatic brain injury
