# Supplementary material for: A Generic Multi-Compartmental CNS Distribution Model Structure for 9 Drugs Allows Prediction of Human Brain Target Site Concentrations
Source: Pharm Res. 2016 Nov 18;34(2):333–51. doi: 10.1007/s11095-016-2065-3 (PMC5236087; doi:10.1007/s11095-016-2065-3)
Supplement: Supplementary file 3 — (DOCX 33 kb) [file 11095_2016_2065_MOESM3_ESM.docx]

**Table SI**. Physicochemical properties of the 9 compounds

|  | Molecular weight | PSA | log P | log D  (pH 7.4) | No. of H donors | No. of H acceptors | pKa  (Acid) | pKa  (Base) |
| --- | --- | --- | --- | --- | --- | --- | --- | --- |
| Acetaminophen | 151 | 49 | 0.5 | 0.5 | 2 | 2 | 9.5 | -4.4 |
| Atenolol | 266 | 85 | 0.2 | -1.7 | 3 | 4 | 14.1 | 9.7 |
| Methotrexate | 454 | 211 | -1.9 | -2.5 | 5 | 12 | 3.4 | 2.8 |
| Morphine | 285 | 53 | 0.9 | -0.4 | 2 | 4 | 10.3 | 9.1 |
| Paliperidone | 426 | 82 | 1.8 | 2.1 | 1 | 5 | 13.7 | 8.8 |
| Phenytoin | 252 | 58 | 2.47 | 2.5 | 2 | 2 | 9.47 | -9 |
| Quinidine | 324 | 46 | 3.4 | 2.0 | 1 | 4 | 13.9 | 9.1 |
| Remoxipride | 371 | 51 | 2.1 | 0.7 | 1 | 4 | 13.1 | 8.4 |
| Risperidone | 410 | 62 | 2.5 | 2 | 0 | 4 |  | 8.8 |
| from DrugBank (http://www.drugbank.ca/) | | | | | | | | |
