# Supplementary material for: A Generic Multi-Compartmental CNS Distribution Model Structure for 9 Drugs Allows Prediction of Human Brain Target Site Concentrations
Source: Pharm Res. 2016 Nov 18;34(2):333–51. doi: 10.1007/s11095-016-2065-3 (PMC5236087; doi:10.1007/s11095-016-2065-3)
Supplement: Supplementary file 4 — (DOCX 38 kb) [file 11095_2016_2065_MOESM4_ESM.docx]

**Table SII**. Summary of the *in vivo* experimental setting in rat data

|  |  |  | Model development | | | | | | | | | |  | External validation | |
| --- | --- | --- | --- | --- | --- | --- | --- | --- | --- | --- | --- | --- | --- | --- | --- |
|  |  |  | Published data | | | | | | | Newly produced data | | |  | Published data | Newly produced data |
| **Study design** | |  | **Acetaminophen** | **Atenolol** | **Methotrexate** | **Morphine** | **Morphine** | **Quinidine** | **Remoxipride** | **Paliperidone** | **Phenytoin** | **Risperidone** |  | **Acetaminophen** | **Remoxipride** |
| samples plasma sampling points | |  | -5, 2, 7, 10, 15, 30, 60, 120, 180, 240 min | 0, 5, 15, 30, 45, 60, 90, 120 min | -5, 2, 7, 9, 10, 12, 17, 30, 90, 180, 300 min | 15 samples up to 360 min | 0, 8, 20, 70,130, 190 min | -5, 2, 7, 10, 12, 17, 30, 60, 140, 240, 360 min | -5, 5, 10, 20, 35, 60, 90, 120, 150, 240 min | -15, 10/20, 30, 60, 90,120, 180, 240, 300, 360 min | 10 samples up to 480 min | -15, 10/20, 30, 60, 90,120, 180, 240, 300, 360 min |  | 0, 5, 10, 20, 35, 60, 90, 120, 150, 180 min | 0, 2, 7, 10, 16, 22, 40, 150 and 240 min |
| dialysate sampling points | |  | every 10 min up to 120 min, every 20 min from 120 to 240 min | up to 120 min | every 10 min up to 300 min | 25 and 30 dialysate samples up to 360 min | every 5 min during infusion, 10 min from 10 to 60 min, every 20 min from 60 to 180 min | every 10 min up to 240 min, every 20 min from 240 to 360 min | every 10 min up to 120 min, every 20 min from 120 to 240 min | every 20 min up to 240 min | every 10 min up to 480 min | every 20 min up to 240 min |  | every 10 min up to 60 min, every 20 min from 60 to 180 min | every 20 min up to 240 min |
| DEC approval number | |  | 07068 | 02112 | 10094 | 03008 | n.a. (from literature) | 07142 | 06132 | 12049 | 11092 | 12049 |  | 06023 | 13186 |
| **Microdialysis setting** | | | | | | | | | | | | | | | |
| probe material | |  | A | B | C | D | n.a. (from literature) | C | A | C | C | C |  | A | C |
| probe supplier | |  | E | F | E | E | E | E | E | E | E | E |  | E | E |
| PF | |  | G | H | G | H | H | G | H | H | H | H |  | H | H |
| flow rate | |  | 2 uL/min | 7 uL/min | 2 uL/min | 2 uL/min | 2 uL/min | 2 uL/min | 2 uL/min | 1 uL/min | 2 uL/min | 1 uL/min |  | 2 uL/min | 1 uL/min |
| f_P_ (%) | |  | 81% | 91% | 45% | 83% | 83% | 14% | 74% | 8% | 9% | 7% |  | 81% | 74% |
| *in vivo* recovery (loss %) | |  | Brain_ECF_: 12.0 %, CSF_LV_: 8.10%, CSAF_CM_: 8.60 % | Brain_ECF_: 13.0 % | Without probenecid:  Brain_ECF_: 22.1 %, CSF_LV_: 28.1%, CSAF_CM_: 35.9 % With probenecid: Brain_ECF_: 7.10 %, CSF_LV_: 16.9%, CSAF_CM_: 21.6 % | Brain_ECF_ (4mg/kg_)_:16.1 %,  Brain_ECF_ (40mg/kg): 20.3 % | Plasma: 40.9 %, Brain_ECF_: 8.40 % | Brain_ECF_: 9.10 %, CSF_LV_: 2.90%, CSAF_CM_: 3.50 % | Brain_ECF_: 20 % | Brain_ECF_: 18.0 %, CSAF_CM_: 10.0 % | Brain_ECF_: 35.0 % | Brain_ECF_: 22.0 %, CSAF_CM_: 10.0 % |  | Brain_ECF_: 12.0 %, CSF_LV_: 8.10%, CSAF_CM_: 8.60 % | Brain_ECF_:11.0 %, CSF_LV_: 7.00 %, CSF_CM_: 5.00 % |
| *in-vivo* recovery references | |  | Westerhout, 2011 | de lange, 1994 | Westerhout, 2014 | Groenendaal, 2007 | Bouw, 2000 | Westerhout, 2013 | Chaurasia, 2007 |  |  |  |  | Westerhout, 2011 |  |
| f_P_ references | |  | Westerhout, 2011 | Srikanth 2013 | Westerhout, 2014 | Stain, 1995 | Stain, 1995 | Westerhout, 2013 | Widman, 1993 |  | Srikanth 2013 |  |  | Westerhout, 2011 | Widman, 1993 |

A; Polycarbonate, B; Cellulose acetate, C; Polyarylethersulphone (PAES), D; polycarbonate-poly-ether (PC-PE) co-polymeric membrane, E; CMA Microdialysis AB, Kista, Sweden, F; Home made, G; 140.3 mM sodium, 2.7 nM potassium, 1.2 mM calcium, 1.0 mM magnesium and 147.7 mM chloride, H; NaCl 145 mM, KCl 0.6 mM, MgCl_2_ 1.0 mM, CaCl_2_ 1.2 mM and ascorbic acid 0.2 mM in 2 mM phosphate buffer (pH 7.4)

PF: microdyalysis perfusion fluid

f_P_: free fraction in plasma
