# Supplementary material for: A Generic Multi-Compartmental CNS Distribution Model Structure for 9 Drugs Allows Prediction of Human Brain Target Site Concentrations
Source: Pharm Res. 2016 Nov 18;34(2):333–51. doi: 10.1007/s11095-016-2065-3 (PMC5236087; doi:10.1007/s11095-016-2065-3)
Supplement: Supplementary file 5 — (DOCX 34 kb) [file 11095_2016_2065_MOESM5_ESM.docx]

**Table SIII**. Demographic data of the patients in the acetaminophen study (study 1)

| Patient | 1 | 2 | 3 | 4 | 5 | 6 | 7 |
| --- | --- | --- | --- | --- | --- | --- | --- |
| Gender | M | F | F | F | F | M | F |
|  | | | | | | | |
| age (year) | 51 | 49 | 76 | 62 | 54 | 79 | 42 |
| height (cm) | 178 | 170 | 160 | 155 | 160 | 175 | 168 |
| weight (kg) | 64 | 65 | 56.5 | 70 | 56 | 74 | 57 |
| number of doses before inclusion | 43 | 22 | 14 | 29 | 5 | 18 | 25 |
| reason of admission | SAH | SAH | SAH | SAH | SAH | SAH | SAH |
| SAH; subarachnoid hemorrhage | | |  |  |  |  |  |
| M:male, F:female |  |  |  |  |  |  |  |
