# Supplementary material for: A Generic Multi-Compartmental CNS Distribution Model Structure for 9 Drugs Allows Prediction of Human Brain Target Site Concentrations
Source: Pharm Res. 2016 Nov 18;34(2):333–51. doi: 10.1007/s11095-016-2065-3 (PMC5236087; doi:10.1007/s11095-016-2065-3)
Supplement: Supplementary file 6 — (DOCX 40 kb) [file 11095_2016_2065_MOESM6_ESM.docx]

**Table SIV**. Acetaminophen EVD experimental data

|  | Patient 1 | |  |  | Patient 5 | |
| --- | --- | --- | --- | --- | --- | --- |
|  | V_EVD_ | Q_LV_EVD_ |  |  | V_EVD_ | Q_LV_EVD_ |
|  | (mL) | (mL/h) |  |  | (mL) | (mL/h) |
| Baseline | 4 | 4 |  | baseline | 21 | 21 |
| 0-0.5h | 4 | 8 |  | 0-1h | 11 | nd |
| 0.5-1h | 4 | 8 |  | 1h-1.5 | 5 | 10 |
| 1h-1.5h | 5 | 10 |  | 1.5-2h | 5 | 10 |
| 1.5-2h | 8.5 | 17 |  | 2-2.5h | 5.5 | 11 |
| 2-2.5h | 1.5 | 3 |  | 2.5-3h | 8 | 16 |
| 2.5-3h | 4 | 8 |  | 3-4h | 14 | 14 |
| 3-3.5h | 3.5 | 7 |  | 4-5h | 14 | 14 |
| 3.5-4h | 7 | 7 |  | 5-6h | 7 | 7 |
| 4-5h | 2 | 2 |  |  |  |  |
| 5-6h | 13 | 13 |  |  |  |  |
| 6-7h | 3 | 3 |  |  | Patient 6 | |
| 7-8h | 7 | 7 |  |  | V_EVD_ | Q_LV_EVD_ |
|  |  |  |  |  | (mL) | (mL/h) |
|  | Patient 2 | |  | baseline | 9 | 9 |
|  | V_EVD_ | Q_LV_EVD_ |  | 0-1h | 5.5 | nd |
|  | (mL) | (mL/h) |  | 1h-1.5 | 6 | 12 |
| Baseline | 18 | 9 |  | 1.5-2h | 0.75 | 1.5 |
| 0-0.5h | 6.5 | 13 |  | 2-2.5h | 4.5 | 9 |
| 0.5-1h | 5 | 10 |  | 2.5-3h | 3 | 6 |
| 1h-1.5h | 5 | 10 |  | 3-4h | 7.5 | 7.5 |
| 1.5-2h | 3 | 6 |  | 4-5h | 8 | 8 |
| 2-3h | 12 | 24 |  | 5-6h | 12 | 12 |
| 3.0-4h | 15 | 30 |  |  |  |  |
| 4-5h | 10 | 10 |  |  |  |  |
| 5-6h | 15 | 15 |  |  | Patient 7 | |
| 6-7h | 10 | 10 |  |  | V_EVD_ | Q_LV_EVD_ |
| 7-8h | 6 | 6 |  |  | (mL) | (mL/h) |
|  |  |  |  | baseline | 12 | 12 |
|  | Patient 3 | |  | 0-1h | 4.75 | nd |
|  | V_EVD_ | Q_LV_EVD_ |  | 1h-1.5 | 4.6 | 9.2 |
|  | (mL) | (mL/h) |  | 1.5-2h | 0.75 | 1.5 |
| Baseline | 17 | 17 |  | 2-2.5h | 2.75 | 5.5 |
| 0-1h | 8 | 8 |  | 2.5-3h | 5.5 | 11 |
| 1-2h | 13 | 13 |  | 3-4h | 17 | 17 |
| 2-3h | 4 | 4 |  | 4-5h | 13 | 13 |
| 3-4h | 12 | 12 |  | 5-6h | 14 | 14 |
| 4-5h | 6 | 6 |  |  |  |  |
| 5-6h | 15 | 15 |  |  |  |  |
|  |  |  |  |  |  |  |
|  |  |  |  |  |  |  |
|  | Patient 4 | |  |  |  |  |
|  | V_EVD_ | Q_LV_EVD_ |  |  |  |  |
|  | (mL) | (mL/h) |  |  |  |  |
| baseline | 14.5 | 14.5 |  |  |  |  |
| 0-1h | 12 | nd |  |  |  |  |
| 1h-1.5 | 4 | 8 |  |  |  |  |
| 1.5-2h | 6.5 | 13 |  |  |  |  |
| 2-2.5h | 1.6 | 3.2 |  |  |  |  |
| 2.5-3h | 6.5 | 13 |  |  |  |  |
| 3-4h | 19 | 19 |  |  |  |  |
| 4-5h | 16.5 | 16.5 |  |  |  |  |
| 5-6h | 16 | 16 |  |  |  |  |
|  |  |  |  |  |  |  |
| nd:no data available. | |  |  |  |  |  |
